# pH-responsive biomimetic zeolitic imidazolate framework-based nanoparticles for co-delivery of cetuximab and siRNA in synergistic therapy of laryngeal squamous cell carcinoma

**DOI:** 10.1016/j.jpha.2025.101203

**Published:** 2025-01-23

**Authors:** Liyin Wang, Milad Ashrafizadeh, Gautam Sethi, Xinjia Zhou

**Affiliations:** a Department of Otolaryngology Head and Neck Surgery, Shengjing Hospital of China Medical University, Shenyang, 110004, China; b Department of Radiation Oncology, Shandong Provincial Key Laboratory of Radiation Oncology, Shandong Cancer Hospital and Institute, Shandong First Medical University, Shandong Academy of Medical Sciences, Jinan, 250000, China; c Department of Pharmacology and NUS Centre for Cancer Research (N2CR), Yong Loo Lin School of Medicine, National University of Singapore, Singapore, 117599, Singapore

**Keywords:** Biomimetic nano co-delivery system, Zeolitic imidazolate framework-8, Cetuximab, siFAK, Laryngeal squamous cell carcinoma

## Abstract

Suboptimal treatment of laryngeal squamous cell carcinoma (LSCC) provides poor survival rate. The poor bioavailability, resistance to cetuximab (Cet), and the instability of small interfering RNA (siRNA) limit their efficacy in LSCC therapy. The present study has been aimed to develop a Cet and focal adhesion kinase (FAK) siRNA (siFAK) co-delivery nanosystem. Zeolitic imidazolate framework-8 (ZIF-8), with its large specific surface area and pH-responsive properties, is an ideal delivery carrier allowing controlled drug release in the acidic tumor microenvironment. Therefore, Cet was loaded onto ZIF-8 and encapsulated in a TU177 cell membrane (TCM) after the electrostatic adsorption of siFAK. Fourier transform infrared (FTIR) spectroscopy, transmission electron microscopy (TEM), scanning electron microscopy (SEM), zeta potential, X-ray diffraction, and particle size analyses were deployed to characterize Cet/siFAK@ZIF-8@TCM. TU177 cells and subcutaneously transplanted tumor-bearing nude mice were used to evaluate the intracellular uptake, cytotoxicity, *in vivo* biocompatibility, biodistribution, biosafety, pH responsiveness, and anti-LSCC efficacy of Cet/siFAK@ZIF-8@TCM. After ZIF-8@TCM were loaded with Cet and siFAK, alterations in their physical and crystal structures, particle size, and zeta potential were observed. Meanwhile, the co-delivery system increased the loading of Cet through the electrostatic adsorption of siFAK to Cet-loaded ZIF-8. The intracellular uptake of Cet/siFAK@ZIF-8@TCM also protected siFAK from degradation, effectively decreasing the messenger RNA (mRNA) and protein expression levels of FAK in LSCC cells. The ZIF-8@TCM nanosystem for co-delivery of Cet and siFAK exhibited pH-responsiveness and tumor-targeting capabilities, thereby exerting anti-LSCC effects. Co-delivery of Cet and siFAK via the pH-responsive ZIF-8@TCM system enabled the targeted release of the chemotherapeutic and gene, in turn maximizing their anti-LSCC effect while ensuring biosafety.

## Highlights

• This study constructed a ZIF-8-based nanosystem for co-delivery Cet and siFAK.
• The nanoparticles modified with TCM exhibited enhanced targeting properties.
• The drug release of Cet/siFAK@ZIF-8@TCM nanoparticles was pH-responsive.
• Cet/siFAK@ZIF-8@TCM nanoparticles exert anti-LSCC activity *in vivo* and *in vitro*.

## Graphical abstract

**Figure:**
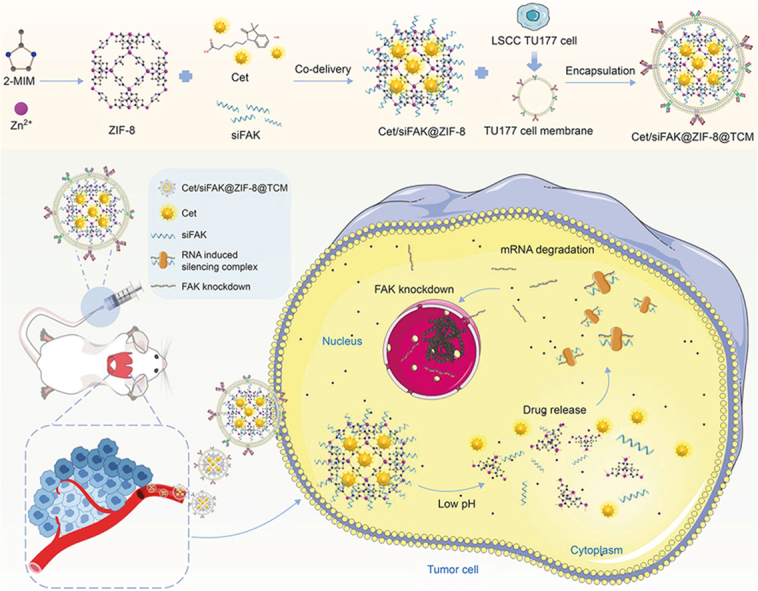

## 1 Introduction

Following lung cancer, laryngeal cancer is the most common neoplasm of upper respiratory tract malignancies, accounting for approximately 20% of head and neck tumors, among which laryngeal squamous cell carcinoma (LSCC) is the most subtype [1]. Epidemiological studies estimate 177,000 new cases of LSCC and 95,000 deaths annually worldwide, with approximately 60% of patients being diagnosed at an advanced stage (lack of specific symptoms in the early stages), increasing death rates [2]. Unfortunately, LSCC is the only subtype of head and neck cancer with a reduced five-year survival rate (from 66% to 63%) [3]; hence, targeted and selective therapeutic strategies, in addition to traditional chemotherapy, should be introduced for its treatment. Cetuximab (Cet), a monoclonal antibody targeting the epidermal growth factor (EGF) receptor (EGFR), is the first molecularly targeted drug approved for use in advanced head and neck squamous cell carcinoma (HNSCC). It is usually utilized in combination with radiotherapy or cytotoxic chemotherapeutic agents to obtain superior therapeutic outcomes by enhancing antigen presentation [[4], [5], [6]]. Clinical data have demonstrated that patients with localized and recurrent/metastatic HNSCC treated with Cet have median survival times of 49 and 10.1 months, respectively [7]. However, these data are unsatisfactory because of the chemical and physical instability, poor bioavailability, resistance, and toxicity of the drug, restricting the therapeutic index, requiring the development of nanoparticles for the targeted drug delivery [8]. Hence, it is suggested to employ targeted delivery systems.

Metal-organic frameworks are promising candidates for the drug delivery systems that can serve as carriers to achieve chemical coupling or physical encapsulation of drugs [9]. Among them, zeolitic imidazolate framework-8 (ZIF-8), which is composed of zinc ions and 2-methylimidazole, demonstrates structural and biological properties including high porosity, ease of modification, biocompatibility, and biodegradability [10]. Notably, ZIF-8 nanoparticles are relatively stable under physiological conditions but demonstrate degradation in the acidic environments, making them more suitable for constructing a pH-sensitive delivery system to achieve drug release in the acidic microenvironment of tumors [11]. Liu et al. [12] attached Cet to a ZIF doped with norbornene-modified imidazole, thereby selectively delivering ribonuclease A (RNase A) to the tumor cells overexpressing EGFR. In addition to chemotherapeutic agents, ZIF-8 can efficiently load and exogenously deliver enzymes, small interfering RNA (siRNA), RNA/protein complexes, and plasmids for improved cancer therapy [13,14]. Zhang et al. [15] prepared ZIF-8 granules loaded with polydopamine and ubiquitin-specific protease 30 inhibitors, which effectively suppressed the viability of oral squamous cell carcinoma and depletion of glutamine both *in vitro* and *in vivo*. However, unmodified ZIF-8 nanoparticles are easily trapped in the endothelial system and cleared by the immune system; these restrictions can be addressed by membrane camouflage [16]. Specifically, tumor cell membranes have the advantage of mimicking cancer cell behavior, evading immune responses, and maintaining homotypic cell adhesion [17,18], thereby permitting targeted delivery of drugs to the tumor region. As a result, it is suggested to modify the nanoparticles, especially ZIF-8 nanostructures with the cell membranes in improving their characteristics and application in cancer therapy.

siRNA can regulate expression of specific genes through post-transcriptional gene silencing and it is a promising therapeutic strategy for cancer [19]. In order to improve the potential in tumor suppression, the nanoparticles for the co-delivery of siRNA and drug have been introduced. The co-delivery of VEGF siRNA and phenethyl isothiocyanate via lipid nanoparticles enhanced anti-angiogenic efficacy [20]. In addition, the co-delivery of adriamycin and siRNA against multidrug resistance protein 1 by mesoporous silica nanoparticles improved the treatment of oral squamous carcinoma [21]. ZIF-8 has the advantage of being a nanomaterial that efficiently loads siRNA through electrostatic interactions and protects it from nuclease damage and degradation [22]. Saeinasab et al. [23] utilized a ZIF-8 nano platform to deliver siRNA of the small nucleolar RNA host gene 15, which improved its efficacy in targeting prostate cancer. However, a scheme to optimize the therapeutic efficacy of LSCC using a ZIF-8 nano platform equipped with Cet and siRNA has not yet been proposed. Moreover, the potential of ZIF-8 nanoparticles for co-delivery of siRNA with drugs in LSCC suppression has not been fully evaluated, which is the aim of the current study.

**Scheme 1:**
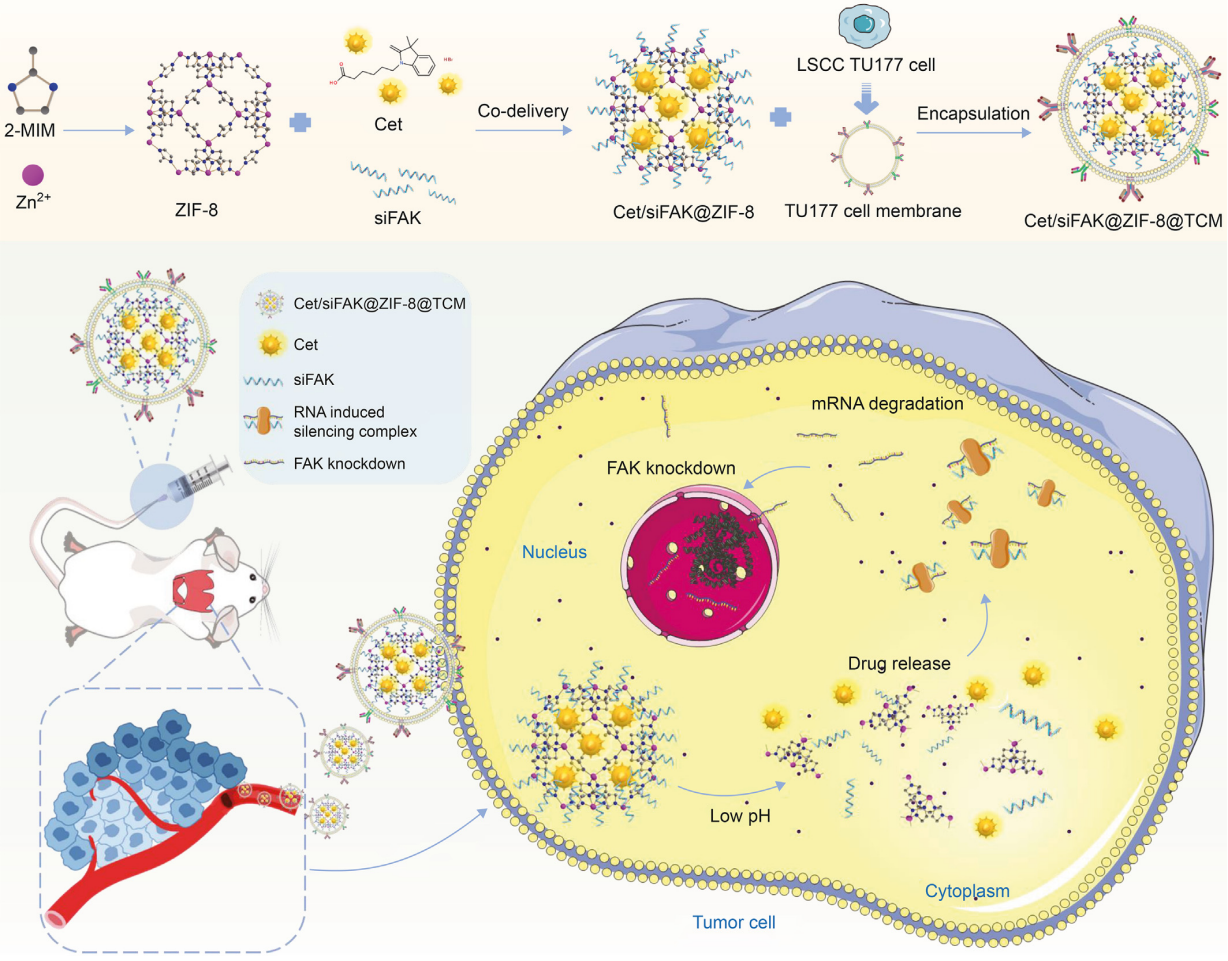
Schematic illustration of the synthesis and application of pH-responsive biomimetic cetuximab (Cet)/focal adhesion kinase (FAK) small interfering RNA (siFAK)@zeolitic imidazolate framework-8 (ZIF-8)@TU177 cell membrane (TCM) for the treatment of laryngeal squamous cell carcinoma (LSCC). 2-MIM: 2-methylimidazole; mRNA: messenger RNA.

According to the previous discussions, we employed pH-responsive ZIF-8 as a nanocarrier for loading Cet, followed by the electrostatic adsorption of focal adhesion kinase (FAK) siRNA (siFAK), to achieve drug delivery and targeted knockdown of oncogene *FAK* expression. To improve the targeting and biocompatibility of Cet/siFAK@ZIF-8, it was encapsulated in the LSCC TU177 cell membrane (TCM), as illustrated in Scheme 1. After characterizing the nanocarriers, we performed *in vivo* and *in vitro* experiments to validate the pH responsiveness, biosecurity, and anti-LSCC efficacy of Cet/siFAK@ZIF-8@TCM. The co-delivery of Cet and siRNA via TCM-camouflaged nanoplatforms provides a constructive approach for exploring synergistic therapies for LSCC. Notably, this study is among the first to evaluate the biomimetic modification of ZIF-8 with a TCM. Furthermore, to the best of our knowledge, this is the first study to evaluate the co-delivery of Cet and siFAK with ZIF-8 for the treatment of LSCC.

## 2 Materials and methods

### 2.1 Materials

Cet (C302740-1 mg) was obtained from Aladdin (Shanghai, China), and ZIF-8 (R-ZF001) was obtained from Xi'an Ruixi Biological Technology Co., Ltd. (Xi'an, China). Methanol (10014108), trichloromethane (10006818), and isopropanol (40064360) were purchased from Sinopharm Chemical Reagent Co., Ltd. (Shanghai, China). Radioimmunoprecipitation assay (RIPA) lysis buffer (P0013B), phenyl methanesulfonyl fluoride (PMSF) (ST506), and 4% paraformaldehyde (PFA) fixation solution (P0099) were acquired from Beyotime Biotechnology (Shanghai, China). Masson's Trichrome Stain Kit (G1340), Triton X-100 (T8200), and Mayer's hematoxylin stain solution (G1080) were obtained from Solarbio Life Sciences (Beijing, China). The siFAK sequences (sense strand (SS) sequence, CGAUUAUAUGUUAGAGAUAGC; antisense strand (AS) sequence, UAUCUCUAACAUAUAAUCGCU) were designed using the designer of siRNA online tool (https://www.invivogen.com/sirnawizard).

### 2.2 Preparation of TCM

Using a Membrane and Cytosol Protein Extraction Kit (P0033; Beyotime Biotechnology), 2 × 10^7^–5 × 10^7^ LSCC TU177 cells were collected for TCM extraction. Briefly, cultured cells were counted and treated with a digestive solution containing ethylenediaminetetraacetic acid (EDTA) but without trypsin. After centrifugation, cell precipitates were collected for further application. Before extracting the cell membranes, the cells were resuspended, and a membrane protein extraction reagent with a final concentration of 1 mM PMSF was prepared. The reagent was then added to the cells and homogenized 30–50 times. At this stage, fragmentation was confirmed to be sufficient if 70%–80% of the cells did not have a shiny ring around the nuclei and/or intact cell morphology. The cell homogenate was then centrifuged for 10 min at 700 *g* and 4 °C to collect the supernatant. Subsequently, the cell membrane fragments were precipitated by further centrifugation for 30 min at 14,000 *g* and 4 °C. The supernatant was collected, extraction reagent B was added, and the mixture vigorously shaken and placed in an ice bath. Lastly, 1–2 mg of TCM was collected after centrifugation for 5 min at 14,000 *g* and 4 °C.

### 2.3 Synthesis of a Cet and/or siFAK loaded ZIF-8 nanocomplex

To load Cet, this compound was mixed with ZIF-8 in methanol at a 1:2 ratio. ZIF-8 was then redispersed in methanol for 20 min using ultrasound to dissolve the Cet. The solutions were mixed and stirred at 600 rpm for 12 h at room temperature. Subsequently, the precipitate was collected by centrifugation at 10,000 rpm for 8 min, washed thrice with methanol, and dried under vacuum at 60 °C to obtain Cet@ZIF-8. The eluted supernatant was collected to calculate the drug-loading efficiency (LE).

To load siFAK, ZIF-8 was mixed with 100 nmol siFAK in a 1:1 ratio by shaking at room temperature for 24 h. After centrifugation at 12,000 rpm for 10 min, the final product siFAK@ZIF-8 was obtained.

To further load Cet and siFAK, Cet@ZIF-8 was mixed with 100 nmol siFAK in a 1:1 ratio and the mixture centrifuged as described above to obtain the final product Cet/siFAK@ZIF-8.

### 2.4 Preparation of Cet and/or siFAK loaded ZIF-8@TCM

To encapsulate Cet@ZIF-8, siFAK@ZIF-8, and Cet/siFAK@ZIF-8 into TCM, the products were mixed with TCM at a ratio of 1:2, respectively. After ultrasonication for 2 min and centrifugation at 12,000 rpm for 10 min, the final products of Cet@ZIF-8@TCM, siFAK@ZIF-8@TCM, and Cet/siFAK@ZIF-8@TCM were obtained.

### 2.5 Nanocomplex characterization

To observe the chemical structure of the fabricated nanocomplexes, the samples were mixed with KBr, pressed into pellets at ambient temperature, and scanned by Fourier transform infrared (FTIR) spectroscopy (Spectrum 1000, PerkinElmer, Waltham, MA, USA). The morphologies of ZIF-8, Cet/siFAK@ZIF-8, and Cet/siFAK@ZIF-8@TCM were observed using transmission electron microscopy (TEM) (Tecnai G2 F30, FEI; Hillsboro, OR, USA) at 100 kV and scanning electron microscopy (SEM) (GeminiSEM 300; ZEISS, Jena, Germany) at 8–10 kV. Furthermore, the nanocomplexes were assayed for the particle size, zeta potential, and crystal structure using a MicrotracMRB-SYNC, Bettersize-BeNano 180 Zeta Pro, and an X-ray diffractometer (XRD) (D8 Advance; Bruker, Karlsruhe, Germany), respectively.

### 2.6 Determination of Cet loading

To prepare Cet/siFAK@ZIF-8 and Cet/siFAK@ZIF-8@TCM test solutions, the supernatant was collected after centrifugation and diluted 10-fold. The absorption at 400 nm was then measured to calculate the LE using the formula below:

LE (%) = (*C*_T_−*C*_D_)/*C*_T_ × 100%

where *C*_T_ represents the total amount of Cet initially added and *C*_D_ represents the dissociative amount of Cet in the supernatant.

### 2.7 *In vitro* release of Cet

To observe the drug release properties of Cet/siFAK@ZIF-8 and Cet/siFAK@ZIF-8@TCM, we dispersed the samples in the dialysis bags containing phosphate-buffered saline (PBS) at different pH values (5.5, 6.5, and 7.4). The pH was adjusted using Tris-HCl-pH 8.8 (ST789-100 mL; Beyotime Biotechnology) and Tris-HCl-pH 6.8 (ST768-100 mL; Beyotime Biotechnology) and measured using a pH meter (Mettler Toledo, Zurich, Switzerland). The absorbance of the samples was measured after immersing the dialysis bags in bottles containing PBS for 0, 6, 12, 18, 24, 30, 36, 42, and 48 h. Based on the standard curve, the drug release rate (*m*) was calculated as follows: *m* = *A*/*B*, where *A* represents the Cet concentration in PBS, and *B* represents the initial concentration of Cet loaded in the nanoparticle.

### 2.8 Cell culture

The TU177 cell line was purchased from Shanghai Yaji Biological Technology Co., Ltd. (YS1198C; Shanghai, China) and maintained in Roswell Park Memorial Institute-1640 (RPMI-1640) medium under routine conditions. A normal human bronchial epithelial cell (NHBEC) line was obtained from BLUEFBIO (BFN60810811; Shanghai, China) and cultured in Dulbecco's modified Eagle's medium (DMEM) under the same conditions. Different concentrations (12.5, 25, 50, 100, and 200 μg/mL) of Cet/siFAK@ZIF-8@TCM were prepared and co-cultured with TU177 and NHBEC cells for 24 h.

### 2.9 Cytotoxicity assay

After co-culture with Cet/siFAK@ZIF-8@TCM for 24 h, TU177 and NHBEC were assayed using a Cell Counting Kit-8 (CCK-8) (C0037; Beyotime Biotechnology) according to the manufacturer's instructions, and cell viability was calculated to determine drug toxicity *in vitro*. Briefly, TU177 and NHBEC cells were inoculated in 96-well plates until wall attachment, followed by the addition of Cet/siFAK@ZIF-8@TCM at different concentrations (12.5, 25, 50, 100, and 200 μg/mL). After the incubation for one day, the CCK-8 reaction solution was added, and the absorbance of the sample was measured at 450 nm.

### 2.10 Cellular uptake of nanoparticles

TU177 and NHBEC cells were digested, collected, fixed, and resuspended to prepare cell suspensions. After labelling nanocomplexes (Cet/siFAK@ZIF-8 and Cet/siFAK@ZIF-8@TCM) with fluorescein isothiocyanate (FITC) (ST2065; Beyotime Biotechnology), cells were treated at a concentration of 50 μg/mL. After 1, 2, 4, 8, and 12 h of incubation, the fluorescence intensity of the cells was detected by flow cytometry (CytoFLEX S; Beckman Coulter, CA, USA) and analyzed using ModFit software. Cellular uptake of nanoparticles was then observed using a confocal laser scanning microscope (CLSM) (ULTRAVIEW VOX; PerkinElmer).

### 2.11 Quantitative real-time polymerase chain reaction (qRT-PCR)

To observe the silencing effect of siFAK, treated TU177 cells and tumor tissues were collected and lysed to release total RNA. After reverse transcription, the samples from each group were subjected to PCR. The messenger RNA (mRNA) expression levels of *FAK in vitro* and *in vivo* were determined using glyceraldehyde-3-phosphate dehydrogenase (GAPDH) as an internal reference. The primer sequences used were as follows: FAK-forward, CCAGGGAAATGCAGGGGATT; FAK-reverse, AAGGAGTATGACCCGCCTCT; GAPDH-forward, AGCTTCAGCCCCAGGAAATC; and GAPDH-reverse, GACATACTGCTGGGCCAGTT.

### 2.12 Western blotting

Western blotting was performed to detect the silencing effect of nanocomplexes loaded with siFAK, as well as markers of homologous targeting and immune escape. Briefly, the treated TU177 cells, tumor tissues, TCM, TCM lysate (TCL), and Cet/siFAK@ZIF-8@TCM were fragmented to release the protein. Following quantification, gel electrophoresis and membrane transfer were performed. Anti-FAK (1:1000, AF6397; Affinity, Melbourne, Australia), anti-histone (1:1000, 9715; Cell Signaling Technology, Danvers, MA, USA), anti-CD44 (1:1000, 37,259; Cell Signaling Technology), anti-CD47 (1:1000, 63,000; Cell Signaling Technology), anti-galectin 3 (1:1000, ab53082; Cell Signaling Technology), anti-E-cadherin (1:1000, 4065, Cell Signaling Technology), and anti-GAPDH (1:3000, ab181602; Abcam, Cambridge, UK) antibodies were incubated with the membrane overnight. After further incubation with secondary antibody for 1 h, color development and imaging were performed. ImageJ software was used to obtain grayscale values of the bands and to calculate the relative protein expression of the targets.

### 2.13 *In vitro* evaluation of pH-responsive *ZIF-8* nanocomplexes

CCK-8 was used to detect the effects of free Cet, free siFAK, and nanocomplexes loaded with Cet and/or siFAK on cell viability at pH 6.5 and 7.4. Flow cytometry was used to detect the effects of Cet, siFAK, Cet@ZIF-8@TCM, siFAK@ZIF-8@TCM, and Cet/siFAK@ZIF-8@TCM TU177 on apoptosis under different pH conditions. After labelling cells using Calcein-acetoxymethylester/propidium iodide (calcein-AM/PI) Double Staining Kit (B-CHK103-500T; Biogradetech, Walnut, CA, USA), the live cells with green fluorescence and dead cells with red fluorescence were observed simultaneously at an excitation of 490 ± 10 nm. Dead cells were observed separately under excitation at 545 nm.

### 2.14 Animal experiments

Thirty-nine BALB/c female nude mice (four weeks old weighting 18–20 g) were purchased from the Laboratory Animal Center of Yangzhou University (Yangzhou, China). The mice were given free access to food and water under specific pathogen-free conditions. After one week of adaptation, 24 mice were randomly divided into four groups, and 3 × 10^5^ TU177 cells were injected subcutaneously into the right axilla of each mouse. When the tumor volume reached 100 mm^3^, 20 mg/kg Cet@ZIF-8@TCM, siFAK@ZIF-8@TCM, and Cet/siFAK@ZIF-8@TCM dissolved in PBS was injected into the mice via the tail vein, and the control group was treated with PBS only. Daily recordings of body weight and tumor volume were started after seven days. Three weeks later, the mice were euthanized, and the tumor tissues were removed, weighed, and preserved. This study was approved by the Experimental Animal Ethics Committee of Yangzhou University, China (Approval No.: 202312014).

### 2.15 *In vivo* biocompatibility

Erythrocytes were extracted from whole blood of BALB/c nude mice and rinsed with cold sterile saline until the supernatant was clear. The 300 μL of erythrocyte suspension was thoroughly mixed with different concentration gradients of Cet/siFAK@ZIF-8@TCM (200 μL). The negative control group was supplemented with PBS (0 hemolysis), whereas the positive control group was supplemented with deionized water (100% hemolysis). After incubation at 37 °C for 12 h, the samples were centrifuged for 15 min at 720 *g*, and the absorbance was measured at 540 nm. The hemolysis ratio was calculated as (sample − PBS)/(H_2_O − PBS) × 100%.

### 2.16 *In vivo* biodistribution

Fifteen BALB/c mice were subjected to the carcinogenic treatment using the same method (as shown in Section 2.14). Dimethyl red (DIR) (HY-D1048, MedChemExpress, Rahway, NJ, USA) was dissolved in dimethylsulfoxide (DMSO) (D8371; Solarbio Life Sciences) to prepare 5 mg/mL DIR solution. The nanoparticle solution was mixed with the DIR solution in a 1:1 ratio and stirred away from light for complete combination. When the tumor volume reached approximately 100 mm^3^, DIR-labelled Cet/siFAK@ZIF-8 and Cet/siFAK@ZIF-8@TCM (20 mg/kg) were injected into the mice via the tail vein. After 2, 6, 12, 24, and 48 h, *in vivo* biodistribution of the nanocomplexes was observed using an *In Vivo* Imaging System (Caliper Life Sciences, Boston, MA, USA). After 48 h, the mice were euthanized, and *in vivo* biodistribution of the nanocomplex in the main organs was determined through imaging.

### 2.17 Immunohistochemistry (IHC)

Mouse tumor tissues were collected and prepared into the sliced tissue using a series of methods, including fixation, washing, dehydration, wax dipping, embedding, and sectioning. The slices were successively incubated with anti-Ki67 (1:200, ab15580; Abcam) and a secondary antibody working solution (1:2000, ab6721; Abcam), followed by color development. After staining with hematoxylin, the sections were imaged, and images were recorded using an optical microscope (PerkinElmer).

### 2.18 Terminal deoxynucleotidyl transferase (TdT)-mediated dUTP nick-end labelling (TUNEL)

The prepared tumor tissue sections were dewaxed and hydrated. After digestion with 50 μL of proteinase K, 5 μL of TdT enzyme, 45 μL of fluorescent labelling solution, and 50 μL of TUNEL assay solution were added to each sample, following the introduction of TUNEL kit (C1091; Beyotime Biotechnology). After incubation for 1 h in the dark, the sections were sealed with an anti-fluorescence quenching agent (p0126; Beyotime Biotechnology) and visualized under a fluorescence microscope (PerkinElmer).

### 2.19 *In vivo* biosafety

Serum samples were prepared from fresh blood of BALB/c mice and assayed for liver (aspartate transaminase (AST), alanine aminotransferase (ALT), and alkaline phosphatase (ALP)) and kidney (serum creatinine (Cre) and blood urea nitrogen (BUN)) function using an automatic biochemical analyzer (3500; HITACHI, Tokyo, Japan). A hematoxylin and eosin (H&E) staining kit (C0105S; Beyotime Biotechnology) was used to detect pathological damage to the heart, liver, spleen, lungs, and kidneys of each group. Briefly, tissues from each group were sectioned and subjected to routine dewaxing and hydration. Subsequently, the sections were stained with hematoxylin solution for 5 min. After cleaning and dehydration, the eosin dye solution was added for 1–2 min. After dehydration with alcohol, the sections were made transparent and sealed for image acquisition under a microscope (PerkinElmer).

### 2.20 Statistical analysis

All data were expressed as mean ± standard deviation (SD) and processed in GraphPad 7.0. Group differences were compared using a one-way analysis of variance (ANOVA), followed by Tukey's post-hoc test. Comparisons between groups for consecutive days were conducted using a two-way repeated-measures ANOVA, with statistical significance set at *P* < 0.05.

## 3 Results

### 3.1 Formulation and characterization of Cet- and siFAK-loaded ZIF-8

**Fig. 1:**
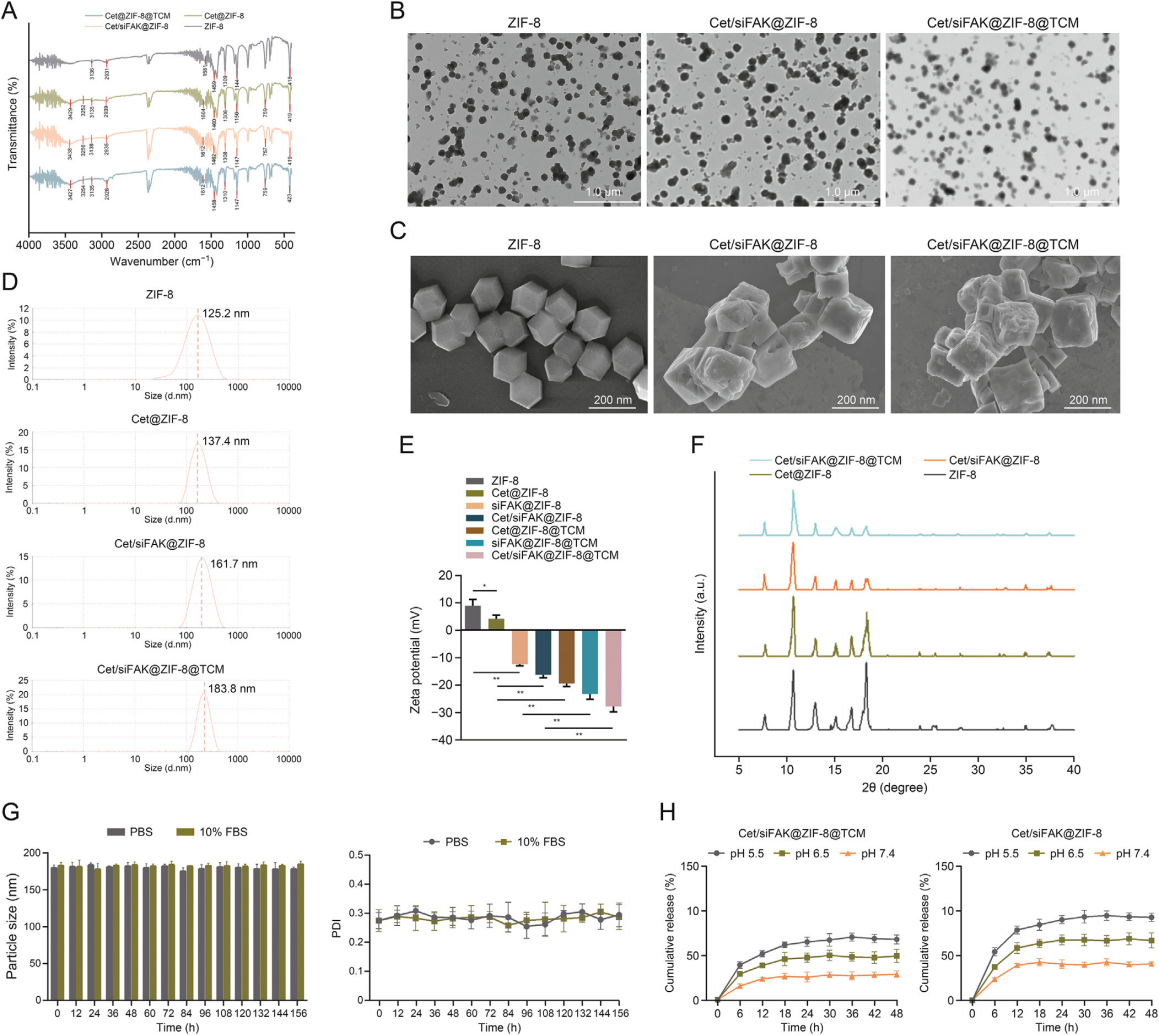
Characterizations of zeolitic imidazolate framework-8 (ZIF-8) loaded with cetuximab (Cet) and/or focal adhesion kinase (FAK) small interfering RNA (siFAK). (A) Characteristic peaks of nanocomplexes in each group obtained using Fourier transform infrared (FTIR) spectroscopy. (B, C) Morphology of ZIF-8 and Cet/siFAK@ZIF-8@TU177 cell membrane (TCM) using transmission electron microscopy (TEM) (B) and scanning electron microscopy (SEM) (C). (D, E) Size distribution by intensity (D) and zeta potential (E) of ZIF-8 loaded with Cet and/or siFAK. ^∗^*P* < 0.05 and ^∗∗^*P* < 0.01. (F) X-ray diffractogram for evaluating the crystallinity of the nanocomplexes. (G) Particle size and polydispersity index (PDI) changes of Cet/siFAK@ZIF-8@TCM in phosphate-buffered saline (PBS) (pH 7.4) or medium containing 10% fetal bovine serum (FBS) at 37 °C for seven days. (H) Cumulative release of Cet from Cet/siFAK@ZIF-8@TCM and Cet/siFAK@ZIF-8 at pH 5.5, 6.5, and 7.4 for two days.

ZIF-8 was used as a carrier to co-deliver Cet and siFAK to achieve sustained drug release and pH responsiveness in the tumor environment. Additionally, TCM was used to encapsulate and camouflage the nanocomplexes to improve their targeting properties. To determine the physical characteristics of the nanocomplexes, FTIR was used, and the characteristic peaks of ZIF-8 and Cet were observed (Fig. 1A). ZIF-8 is composed of zinc ions and 2-methylimidazole, resulting in the appearance of a characteristic C–O absorption peak at 1309 cm^−1^, a weak peak near 2932 cm^−1^ for the stretching vibration of the C–H bond in the imidazole ring, and a weak peak near 3136 cm^−1^ for the stretching vibration of the C–H bond in the methyl ring. Furthermore, the loading of Cet led to the appearance of characteristic peaks at 3252 cm^−1^ for the O–H tensile vibration [24]. TEM and SEM also revealed the dodecahedral morphology of ZIF-8 and an increase in the particle size of Cet/siFAK@ZIF-8 and Cet/siFAK@ZIF-8@TCM, as well as the encapsulation by TCM (Figs. 1B and C).

The particle sizes of ZIF-8, Cet@ZIF-8, Cet/siFAK@ZIF-8, and Cet/siFAK@ZIF-8@TCM were 125.2, 137.4, 161.7, and 183.8 nm, respectively (Fig. 1D), confirming that Cet and siFAK were successfully loaded into ZIF-8 and further encapsulated by TCM. Because of the electrostatic adsorption between positively charged Cet@ZIF-8 and negatively charged siFAK, a significant decrease in the zeta potential of Cet/siFAK@ZIF-8 from 4.13 to −16.3 mV was observed when compared to Cet@ZIF-8 (Fig. 1E). After camouflaging with the negatively charged cell membrane, the potential of the nanocomplex further reduced to −27.8 mV. XRD was used to evaluate the crystallinity of the nanocomplexes, and characteristic peaks at 7.30°, 10.30°, 12.60°, 14.75°, 16.40°, and 17.95° were observed for ZIF-8 (Fig. 1F). Cet@ZIF-8, Cet/siFAK@ZIF-8, and Cet/siFAK@ZIF-8@TCM had crystal structures similar to that of ZIF-8, but the characteristic peaks were smaller. To further evaluate the *in vitro* stability of the nanocomplexes, Cet/siFAK@ZIF-8@TCM was dispersed in PBS and a medium containing 10% fetal bovine serum (FBS), resulting in no significant differences in the particle size and polydispersity index (PDI) of the nanocomplexes within 156 h (Fig. 1G). These results indicate that ZIF-8@TCM loaded with Cet and siFAK was successfully prepared.

### 3.2 Loading and release of Cet

The LE (%) values for Cet/siFAK@ZIF-8 and Cet/siFAK@ZIF-8@TCM were 32.3% and 44.0%, respectively. In simulated physiological environments of pH 5.5, 6.5, and 7.4, drug release rate decreased with increasing pH for both Cet/siFAK@ZIF-8@TCM and Cet/siFAK@ZIF-8 (Fig. 1H). Furthermore, Cet/siFAK@ZIF-8 encapsulated in TCM exhibited a lower rate of Cet release, suggesting the protective and controlled-release properties imparted by the cell membrane.

### 3.3 *In vitro* evaluation of cytotoxicity, homologous targeting, and immune escape

**Fig. 2:**
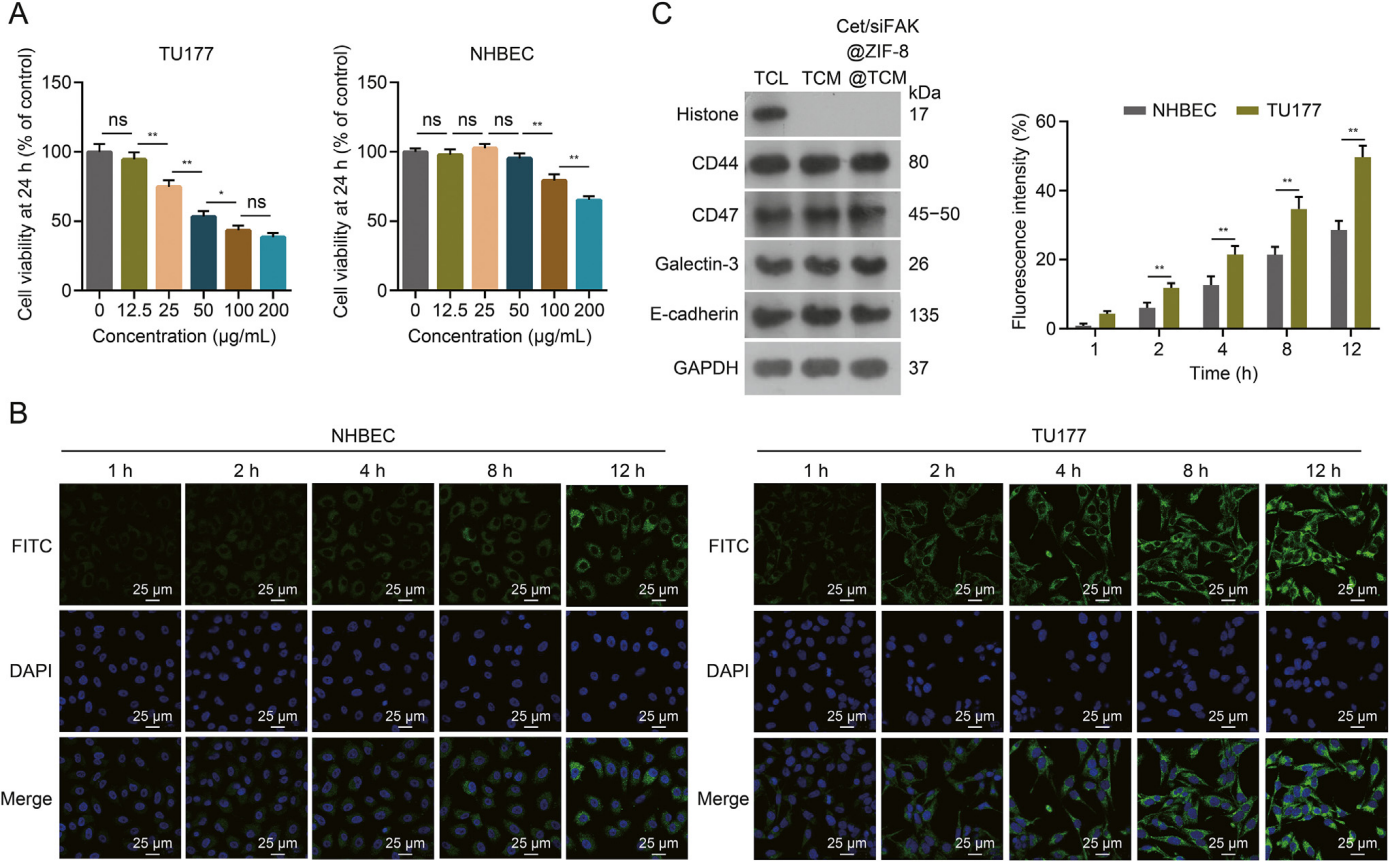
Assessment of cytotoxicity, homologous targeting, and immune escape properties of cetuximab (Cet)/focal adhesion kinase (FAK) small interfering RNA (siFAK)@zeolitic imidazolate framework-8 (ZIF-8)@TU177 cell membrane (TCM) *in vitro*. (A) Changes in viability of TU177 cells and normal human bronchial epithelial cells (NHBECs) at varying concentrations of Cet/siFAK@ZIF-8@TCM. (B) Uptake of Cet-siFAK@ZIF-8@TCM by NHBEC and TU177 cells in the different time periods including 1, 2, 4, 8, and 12 h. (C) Western blotting was used to detect nuclear markers, as well as the homologous targeting and immune evasion properties of tumor cell membranes. ^∗^*P* < 0.05 and ^∗∗^*P* < 0.01. ns: no significance; FITC: fluorescein isothiocyanate; DAPI: 4′,6-diamidino-2-phenylindole; TCL: tumor cell lysate; GAPDH: glyceraldehyde-3-phosphate dehydrogenase.

To assess the toxicity of Cet/siFAK@ZIF-8@TCM *in vitro*, the CCK assay was used to detect the changes in the activity of LSCC cells treated with different concentrations of nanocomplexes. Compared to 12.5 μg/mL, TU177 co-cultured with 25 μg/mL Cet/siFAK@ZIF-8@TCM showed a significant decrease in the cell viability, which was further decreased with the increases in Cet/siFAK@ZIF-8@TCM concentration up to 100 μg/mL (Fig. 2A). In NHBEC cells, cell activity showed no significant inhibition at 0–50 μg/mL Cet/siFAK@ZIF-8@TCM but was significantly suppressed at concentrations of 100 and 200 μg/mL (Fig. 2A). Using CLSM, we found that the uptake of Cet/siFAK@ZIF-8@TCM by TU177 cells was significantly higher than that by NHBEC between 2 and 12 h (Fig. 2B). Furthermore, Western blotting results suggested that the nuclear marker protein histone was only detected in TCL. TCM and Cet/siFAK@ZIF-8@TCM retained functional proteins for immune escape and homologous targeting, including CD44, CD47, galectin-3, and E-cadherin (Fig. 2C). These results suggest that Cet/siFAK@ZIF-8@TCM retains the homologous recognition and immune escape functions of TCM and can better target tumor cells while controlling cytotoxicity.

### 3.4 *In vitro* intracellular uptake

**Fig. 3:**
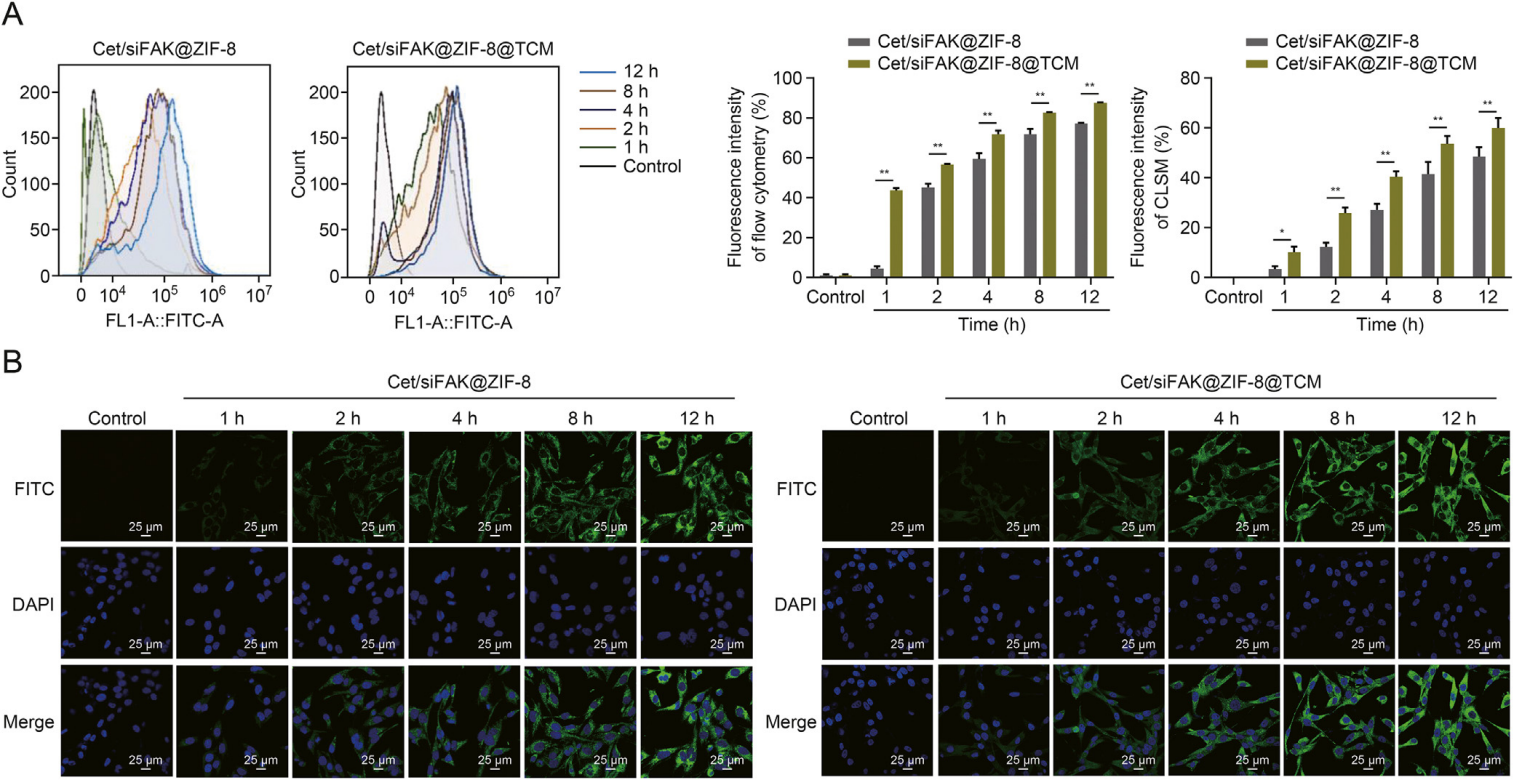
Intracellular uptake of cetuximab (Cet)/focal adhesion kinase (FAK) small interfering RNA (siFAK)@zeolitic imidazolate framework-8 (ZIF-8) and Cet/siFAK@ZIF-8@TU177 cell membrane (TCM) *in vitro* using (A) flow cytometry and (B) a confocal laser scanning microscope (CLSM). ^∗^*P* < 0.05 and ^∗∗^*P* < 0.01. FITC: fluorescein isothiocyanate; DAPI: 4′,6-diamidino-2-phenylindole.

Uptake of Cet/siFAK@ZIF-8@TCM by tumor cells is a prerequisite for anti-LSCC therapy. Therefore, FITC was used to label Cet/siFAK@ZIF-8 and Cet/siFAK@ZIF-8@TCM, and flow cytometry and CLSM were used to observe *in vitro* intracellular uptake of the nanocomplexes. The fluorescence intensity of FITC increased substantially from 0 to 12 h (Fig. 3). More importantly, the uptake of Cet/siFAK@ZIF-8@TCM by the tumor cells was significantly higher than that of Cet/siFAK@ZIF-8. The above results clearly demonstrate that Cet/siFAK@ZIF-8@TCM not only has good biocompatibility but also enables drug accumulation in tumor tissues for enhanced anti-tumor activity.

### 3.5 *In vitro* evaluation on therapeutic efficiency of pH-responsive nanocomplexes

**Fig. 4:**
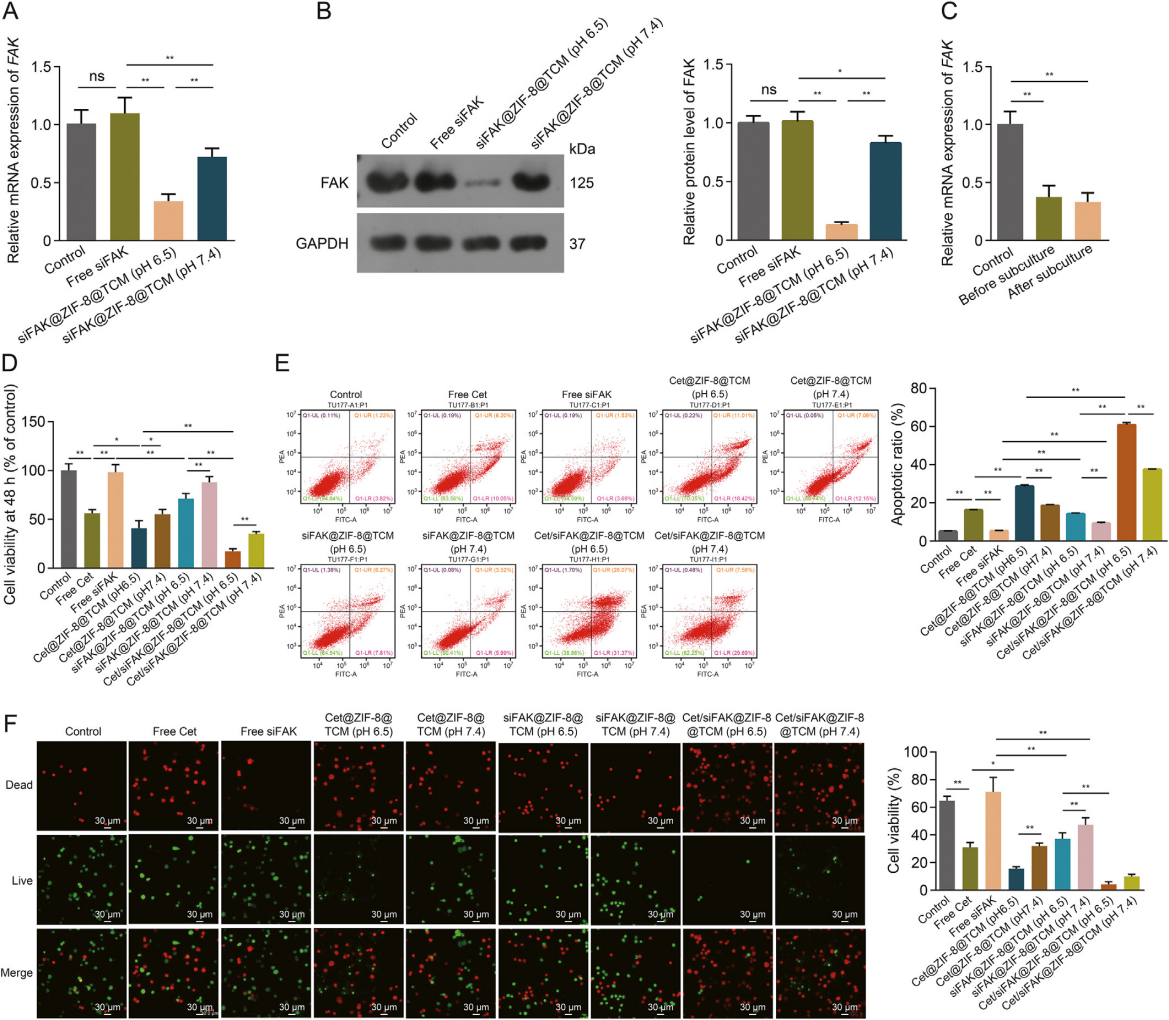
Evaluation of therapeutic efficiency and pH-responsive property of cetuximab (Cet)/focal adhesion kinase (FAK) small interfering RNA (siFAK)@zeolitic imidazolate framework-8 (ZIF-8)@TU177 cell membrane (TCM) *in vitro*. (A, B) The messenger RNA (mRNA) (A) and protein (B) levels of FAK in TU177 cells treated with free siFAK and siFAK-loaded nanocomplexes. (C) FAK mRNA levels of *FAK* in TU177 cells before and after three generations. (D–F) Viability (D), apoptosis (E), and live/dead status (F) of TU177 cells cultured with free Cet, free siFAK, Cet@ZIF-8@TCM, siFAK@ZIF-8@TCM, and Cet/siFAK@ZIF-8@TCM at pH 6.5 and 7.4. ^∗^*P* < 0.05 and ^∗∗^*P* < 0.01. ns: no significance; GAPDH: glyceraldehyde-3-phosphate dehydrogenase; PEA: phycoerythrin-area; UL: upper left; UR: upper right; LL: lower left; LR: lower right; FITC: fluorescein isothiocyanate.

To further confirm the silencing effect of siFAK *in vitro*, PCR and Western blotting experiments were conducted. The results suggested no significant differences in the mRNA and protein levels of FAK in cells treated with free siFAK compared to those in the control (Figs. 4A and B), demonstrating that the addition of free siFAK in the medium did not affect the expression of FAK in cells. However, after co-culture with siFAK@ZIF-8@TCM, both FAK mRNA and protein levels were significantly downregulated (Figs. 4A and B), suggesting that the nanocomplexes loaded with siFAK were successfully taken up by the cells and released siFAK to exert gene-silencing effects. In addition, the *FAK* silencing effect in TU177 cells at pH 6.5 was significantly higher than that at pH 7.5, further confirming the pH-responsiveness of ZIF-8@TCM. In TU177 cells, *FAK* expression was not significantly altered before and after three generations and was significantly lower than that in the control (Fig. 4C), indicating that the nano-loading system can effectively and stably inhibit the expression of *FAK* in cells.

In terms of cell function, TU177 cells cultured with Cet/siFAK@ZIF-8@TCM showed significantly lower viability than free Cet or free siFAK treatment, and the inhibition of cell proliferation was more pronounced at pH 6.5 compared to pH 7.4 (Fig. 4D). Furthermore, Cet@ZIF-8@TCM was better than free Cet, siFAK@ZIF-8@TCM was better than free siFAK, and Cet/siFAK@ZIF-8@TCM was better than Cet@ZIF-8@TCM and siFAK@ZIF-8@TCM in promoting apoptosis. The apoptosis-promoting effect at pH 6.5 was significantly enhanced compared to that at pH 7.4 in all groups (Fig. 4E). Immunofluorescence results showed that the number of TU177 live cells was significantly reduced after treatment with the nanocomplexes when compared to free Cet or siFAK at pH 6.5, with the acidic environment having superior therapeutic effects compared to that at pH 7.4 (Fig. 4F), further confirming that the anti-LSCC effect of pH-responsive Cet/siFAK@ZIF-8@TCM was superior to that of Cet and siFAK alone *in vitro*.

### 3.6 *In vivo* biocompatibility, biodistribution, and siFAK silencing efficiency

**Fig. 5:**
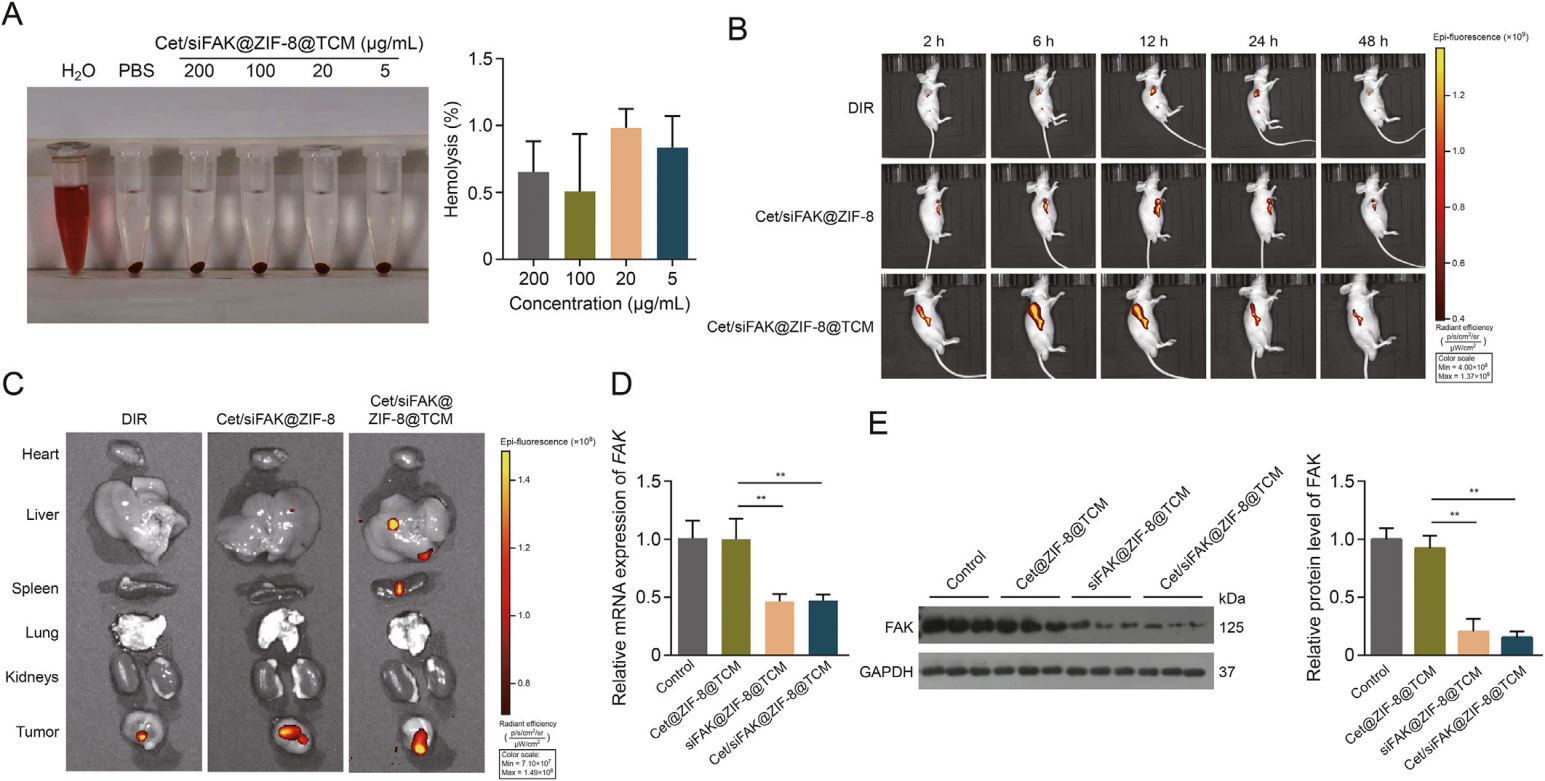
*In vivo* biodistribution of cetuximab (Cet)/focal adhesion kinase (FAK) small interfering RNA (siFAK)@zeolitic imidazolate framework-8 (ZIF-8)@TU177 cell membrane (TCM) and silencing efficiency of siFAK. (A) Hemolysis assay of Cet/siFAK@ZIF-8@TCM at various concentrations. (B) *In vivo* biodistribution of dimethyl red (DIR)-labelled Cet/siFAK@ZIF-8 and Cet/siFAK@ZIF-8@TCM at 2, 6, 12, 24, and 48 h. (C) *In vivo* biodistribution of nanocomplexes in the main organs. (D, E) Messenger RNA (mRNA) (D) and protein (E) levels of FAK in tumor tissues after injecting Cet@ZIF-8@TCM, siFAK@ZIF-8@TCM, and Cet/siFAK@ZIF-8@TCM. ^∗∗^*P* < 0.01. PBS: phosphate-buffered saline; GAPDH: glyceraldehyde-3-phosphate dehydrogenase.

To determine the safety of Cet/siFAK@ZIF-8@TCM for *in vivo* application, a hemolysis assay was conducted. Cet/siFAK@ZIF-8@TCM at concentrations in the range of 5–200 μg/mL exhibited almost no hemolysis (Fig. 5A), making them suitable for intravenous injection. To explore the anti-LSCC effect of Cet/siFAK@ZIF-8@TCM *in vivo*, we labelled the nanocomplex with DIR to observe its biodistribution. *In vivo* imaging indicated that the fluorescence intensity of the labeled Cet/siFAK@ZIF-8@TCM reached a maximum at 6 h after injection into mice, remained at a high level until 12 h, and then gradually weakened (Fig. 5B). The fluorescence intensity of Cet/siFAK@ZIF-8@TCM was significantly higher than that of Cet/siFAK@ZIF-8. Images of the main organs suggested the biodistribution of Cet/siFAK@ZIF-8@TCM in the liver, spleen, and tumor tissues (Fig. 5C). The results illustrate that the modified nanocomplex could prolong the sustained and effective release of the drug into the bloodstream, thereby enhancing the therapeutic effect of Cet. Additionally, ZIF-8@TCM equipped with siFAK significantly reduced the mRNA and protein expression levels of FAK in the tumor tissues compared to the control and Cet@ZIF-8@TCM (Figs. 5D and E).

### 3.7 Anti-LSCC effects of nanocomplexes *in vivo*

**Fig. 6:**
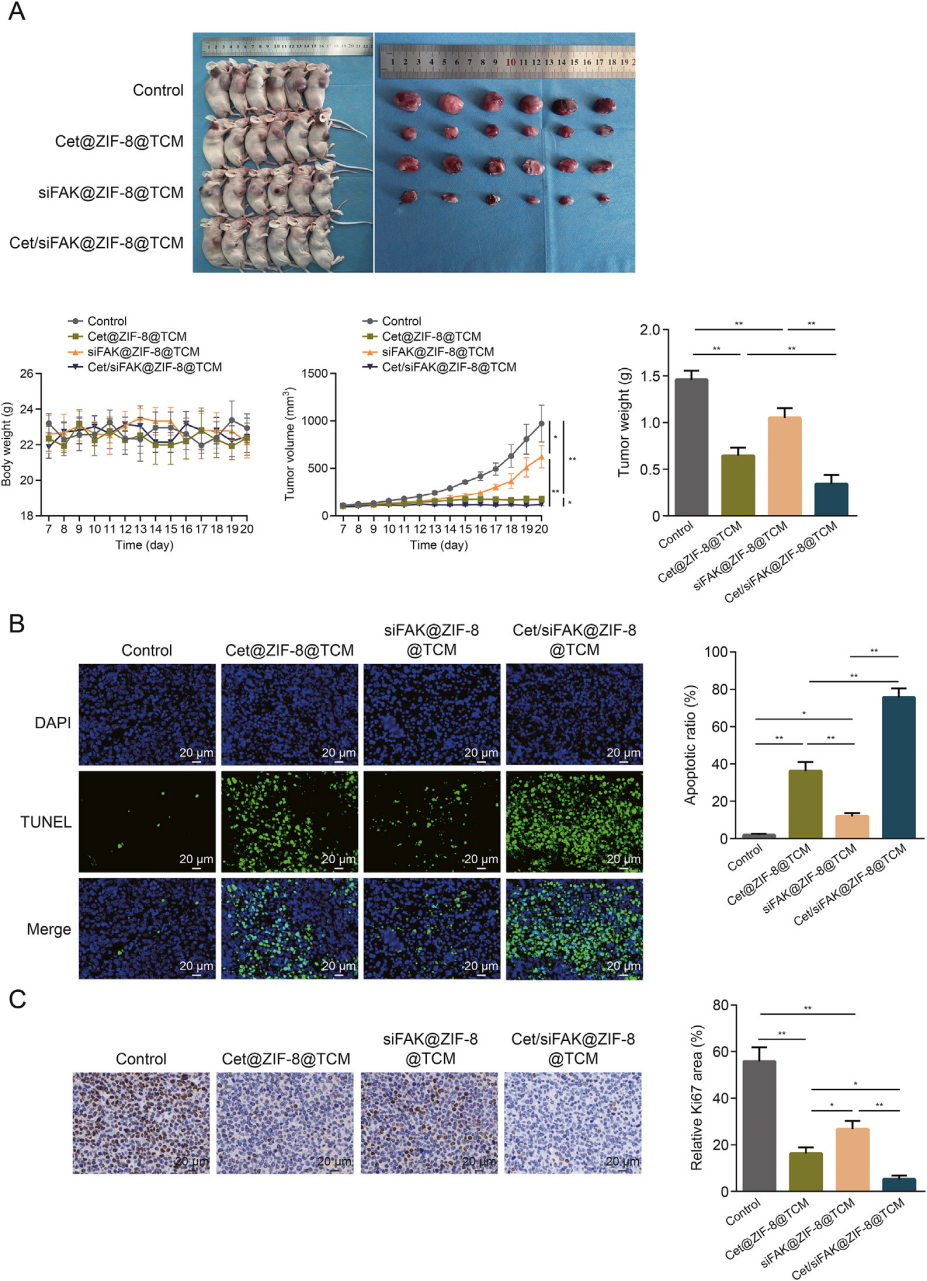
Investigation of anti-laryngeal squamous cell carcinoma (LSCC) effects of cetuximab (Cet)/focal adhesion kinase (FAK) small interfering RNA (siFAK)@zeolitic imidazolate framework-8 (ZIF-8)@TU177 cell membrane (TCM) *in vivo*. (A) Images of mice and tumors from mice injected with Cet@ZIF-8@TCM, siFAK@ZIF-8@TCM, or Cet/siFAK@ZIF-8@TCM (up) and changes in body weight, tumor volume, and tumor weight (down). (B) Terminal deoxynucleotidyl transferase (TdT)-mediated dUTP nick-end labelling (TUNEL) assay (left) and apoptotic ratio (right) for tumor tissues from mice treated with Cet@ZIF-8@TCM, siFAK@ZIF-8@TCM, and Cet/siFAK@ZIF-8@TCM. (C) The immunohistochemistry (IHC) staining (left) and the relative expression (right) of Ki67 in tumor tissues from mice treated with Cet@ZIF-8@TCM, siFAK@ZIF-8@TCM, and Cet/siFAK@ZIF-8@TCM. ^∗^*P* < 0.05 and ^∗∗^*P* < 0.01. DAPI: 4′,6-diamidino-2-phenylindole.

The observation and analysis of the tumor tissues suggested that, compared to the control, Cet@ZIF-8@TCM and siFAK@ZIF-8@TCM significantly inhibited tumor volume and weight, which were further decreased by Cet/siFAK@ZIF-8@TCM treatment (Fig. 6A). After labelling apoptotic cells using the TUNEL assay, we found that Cet/siFAK@ZIF-8@TCM significantly promoted tumor cell apoptosis compared to Cet@ZIF-8@TCM and siFAK@ZIF-8@TCM (Fig. 6B). Ultimately, Ki67, a tumor growth marker, was detected by IHC, and the results suggested that the nanocomplexes significantly inhibited the expression of Ki67 compared to the control group, whereas the inhibitory effect of Cet/siFAK@ZIF-8@TCM was significantly better than that of the other groups (Fig. 6C). These results indicate that Cet/siFAK@ZIF-8@TCM can effectively target LSCC and exert anti-LSCC effects *in vivo*.

### 3.8 Assessment of biosafety *in vivo*

**Fig. 7:**
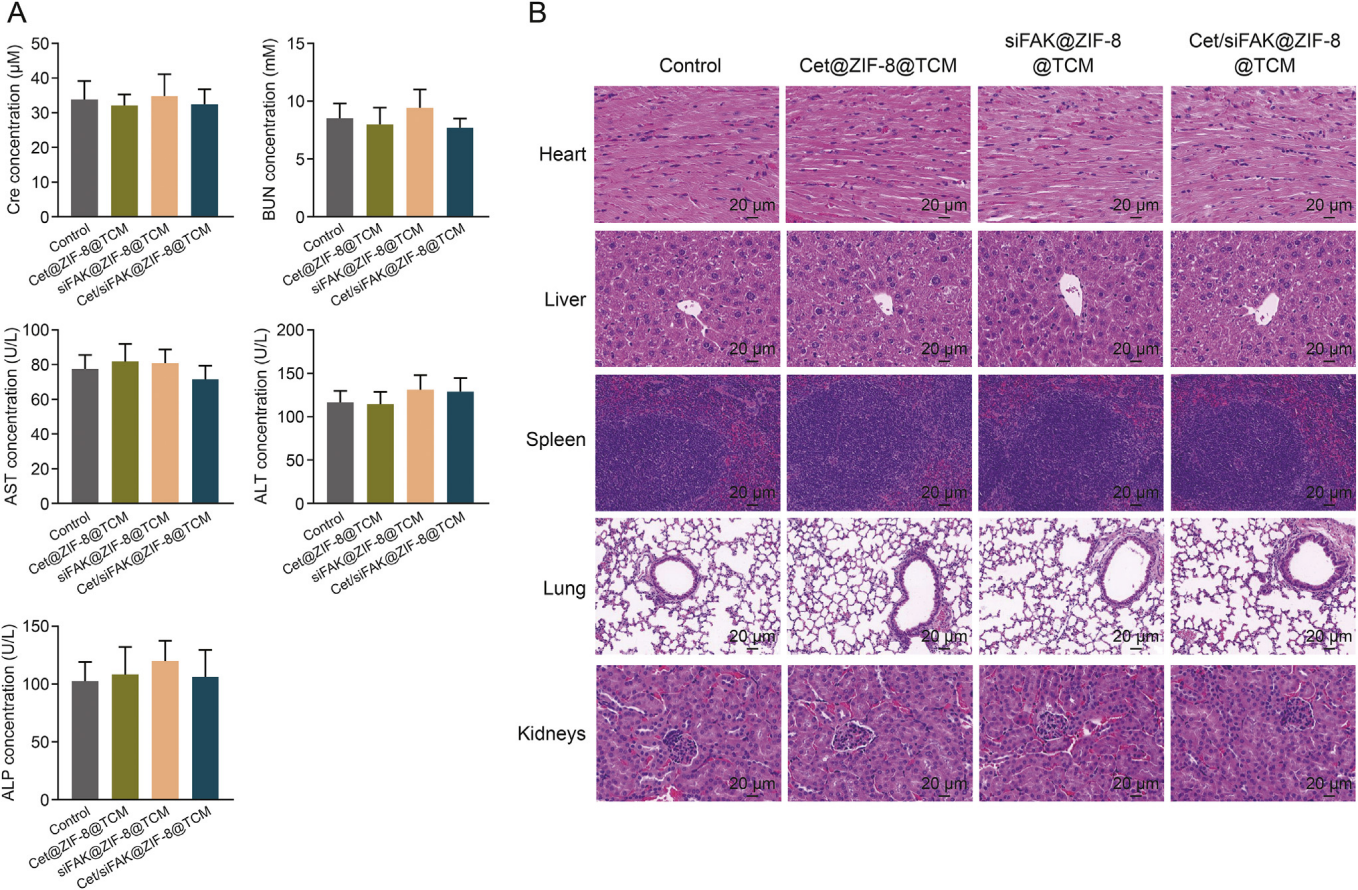
*In vivo* biosafety evaluation. (A) Differences in liver and kidney function indicators between the control, cetuximab (Cet)@zeolitic imidazolate framework-8 (ZIF-8)@TU177 cell membrane (TCM), focal adhesion kinase (FAK) small interfering RNA (siFAK)@ZIF-8@TCM, and Cet/siFAK@ZIF-8@TCM. (B) Hematoxylin and eosin (H&E) staining showing the histological morphology of the main organs. Cre: creatinine; BUN: blood urea nitrogen; AST: aspartate transaminase; ALT: alanine aminotransferase; ALP: alkaline phosphatase.

To estimate the biosafety of the nanocomplexes in mice, serum Cre, BUN, AST, ALT, and ALP levels were measured. No differences in the indicators of liver and kidney function were observed among the control, Cet@ZIF-8@TCM, siFAK@ZIF-8@TCM, and Cet/siFAK@ZIF-8@TCM groups (Fig. 7A). The histological morphology of the main organs was observed by H&E staining. The results revealed that mice treated with Cet@ZIF-8@TCM, siFAK@ZIF-8@TCM, and Cet/siFAK@ZIF-8@TCM did not experience any significant damage to the heart, liver, spleen, lung, or kidney tissues (Fig. 7B), demonstrating that the designed nanoparticles have desirable safety and biocompatibility.

## 4 Discussion

In the present study, a pH-responsive nanosystem that co-delivered Cet and siFAK using TCM encapsulation was designed to achieve the targeted release of drug and knockdown of the pro-carcinogenic gene *FAK* in tumor site. Characterization of the nanocomplex suggested that ZIF-8@TCM loaded with Cet and siFAK exhibited a shift in the characteristic peak and an increase in the particle size. In addition, the physical structure of TCM was observed following TEM and SEM. Regarding to the electrostatic adsorption between the positively charged Cet@ZIF-8 and negatively charged siFAK, there was a significant decrease in the zeta potential of the nanoparticles loaded with siFAK. Furthermore, the drug loading rate of Cet/siFAK@ZIF-8 encapsulated in TCM increased from 32.3% to 44.0%, indicating that TCM protected Cet from exogenous release. These results indicated the successful construction of Cet/siFAK@ZIF-8@TCM.

The pH response of this co-delivery system was mainly attributed to the acid sensitivity of ZIF-8. Under acidic conditions, protonation of the organic ligand of ZIF-8 leads to the breakage of the Zn^+^-imidazolium ion ligand bond, which decomposes the ZIF-8 skeleton to release the drug [25]. The pH differs between normal (7.4) and cancerous (6.5–7) areas [26,27]; hence, the acid sensitivity of ZIF-8 promotes its tumor-targeting ability by releasing its cargo in the acidic tumor microenvironment, improving the specificity and selectivity in targeting cancer cells. An *in vitro* study demonstrated that ZIF-8 nanoparticles released up to 75.9% of the drug at pH 5.5, which was higher compared those at pH 7.4 [28]. Furthermore, ZIF-8 is stable under neutral conditions, but the ligands in the framework dissociate from metal ions in the acidic environments to accelerate drug release [29]. Therefore, the application of ZIF-8 nanostructures is promising for the pH-responsive release of cargo and responding to the specific stimuli in the tumor microenvironment. Herein, the drug release rate of ZIF-8-based nanocomplexes decreased with increasing pH. The pH responsiveness of Cet/siFAK@ZIF-8@TCM was confirmed in the cellular experiments, where the nanocomplexes at pH 6.5 showed more significant inhibition of cell proliferation and promotion of apoptosis compared to those at pH 7.4. Furthermore, nanoparticles encapsulated with TCM exhibited a decreased drug release rate, suggesting that the bionic cell membrane protects ZIF-8 complexes from degradation by acidic tumor substances. Smaller nanoparticles have faster drug release kinetics, regardless of pH, but excessive instantaneous concentrations of drugs may cause uncontrolled cytotoxicity [25]. Cell membrane camouflage of the ZIF-8-based nanoparticles allowed for properties and functions similar to those of source cells, facilitating the aggregation and internalization of homotypic cells [30,31]. Therefore, encapsulation using TCM not only prevented Cet/siFAK@ZIF-8 from being trapped by the immune system, thus directly targeting tumor cells, but also prolonged the circulation time of the drug while preventing cytotoxicity.

Furthermore, this nano-co-delivery system exhibited RNA therapy through targeting and inhibiting the expression of oncogene *FAK*. *FAK* participates in the cell-extracellular matrix interactions and demonstrates upregulation in HNSCC and laryngeal epithelial precursor lesions [32]. In addition, *FAK* contributes to the activation of the EGF signaling pathway and is significantly correlated with an increased risk of laryngeal cancer development and recurrence [1,33]. Therefore, ZIF-8-based nanocomplexes electrostatically adsorbed siFAK to escape from the cytoplasm and avoid siRNA degradation, thereby facilitating the effective delivery of the latter. *In vitro* data also showed that siFAK@ZIF-8@TCM significantly reduced the mRNA and protein expression levels of FAK in TU177 cells compared to free siFAK, thus exerting its antitumor effect. Moreover, Uzawa et al. [34] proposed that acquired resistance to Cet is partly caused by the genetic alterations in patients with oral squamous cell carcinoma. Interestingly, a recent study found that exosomal delivery of *FAK* siRNA reversed Cet resistance in colon cancer [35]. Our *in vivo* experiments further confirmed that Cet/siFAK@ZIF-8@TCM significantly suppressed tumor volume and Ki67 expression, and promoted apoptosis of tumor cells compared to Cet@ZIF-8@TCM and siFAK@ZIF-8@TCM. Hence, nanosystems that co-deliver Cet and siFAK may enhance the drug sensitivity of tumor cells to Cet, thereby maximizing its anti-LSCC effects. Moreover, the co-delivery of Cet and siRNA improved the potential of ZIF-8 nanoparticles for drug loading. Notably, the combination of siFAK and Cet within ZIF-8 improved simultaneous loading of both therapeutic compounds. Consequently, the nanostructure accelerated the electrostatic adsorption of siFAK and Cet-loaded ZIF-8 to develop a stable complex and improve the overall loading ability, as confirmed by the increased LE from 32.3% to 44.0%.

Although the safety and efficacy of Cet/siFAK@ZIF-8@TCM were demonstrated in cell- and animal-based experiments, comprehensive long-term *in vivo* safety data and extensive preclinical studies are required. The membrane may be affected by the biochemical actions, temperature changes, or mechanical stresses under certain conditions, resulting in the loss of integrity and reduced stability of the drug delivery system [9]. In addition, it is important to note that the exogenous supply of Cet/siFAK@ZIF-8@TCM may contain certain components or structures that may induce cytotoxic or inflammatory responses, which were not explored in this study. Lastly, there is still a lack of data to support whether the typical adverse events of Cet-treated LSCC, such as an acne-like skin rash, can be improved using the developed Cet/siFAK@ZIF-8@TCM co-delivery system. Therefore, extensive toxicological evaluations and safety testing of this nano-co-delivery system are necessary before transitioning from laboratory to clinical applications.

From a scalability perspective, the different components used in the nanoparticles, such as ZIF-8, Cet, siFAK, and TCM, are well understood and can be synthesized using chemical and biological methods. This straightforward reaction between zinc nitrate and 2-methylimidazole is both scalable and affordable. Cet is already being utilized in the clinic. The generation of siFAK is based on the standard oligonucleotide synthesis, which can be scaled using current methods. TCM extraction may be optimized for the large-scale production as membrane isolation technology is well understood and can be adapted for further applications in industrial-scale methods. From an economic perspective, the cost of production depends on the synthesis of ZIF-8 and the therapeutic compounds, including Cet and siFAK. The materials deployed for the synthesis of ZIF-8 are affordable and the synthetic process is of low-cost, making it viable for large-scale production. The cost of Cet is remarkable; however, its combination with siFAK and ZIF-8 provides a better platform for the drug delivery, reducing the required dose and overall cost. The generation of siFAK is affordable and can be increased based on the required quantity. In addition, bulk synthesis and advancements in RNA production can lead to cost reduction.

## 5 Conclusions

The purpose of the present study was to develop a biomimetic drug delivery system utilizing pH-responsive ZIF-8 nanoparticles loaded with Cet, electrostatically adsorbed siFAK, and encapsulated in TCM to pave the way in cancer therapy. This co-delivery nanosystem of Cet and siFAK demonstrated enhanced Cet loading and prevented the degradation of siFAK, thereby enabling the targeted release of these agents in tumor tissues and targeted knockdown of the pro-carcinogenic gene *FAK*. *In vivo* and *in vitro* experiments confirmed the pH-responsiveness and biosafety of Cet/siFAK@ZIF-8@TCM and its anti-LSCC progression advantages, thereby providing a new, feasible, and clinically promising drug delivery strategy for the treatment of LSCC. This study highlights the potential of ZIF-8 nanoparticles for co-delivery benefits and also demonstrates the combination of gene therapy and chemotherapy in the suppression of LSCC. In order to improve the release of therapeutics at the tumor site, the nanoparticles were designed to be pH-sensitive. Moreover, TCM modification of nanoparticles can improve their targeted delivery and ability to target cancer cells.

## CRediT authorship contribution statement

**Liyin Wang:** Writing – Original draft, Data curation, Conceptualization. **Milad Ashrafizadeh:** Writing – Review & editing, Writing – Original draft, Validation, Supervision. **Gautam Sethi:** Writing – Review & editing, Writing – Original draft, Supervision, Methodology, Investigation. **Xinjia Zhou:** Writing – Original draft, Validation, Supervision, Software, Resources, Conceptualization.

## Declaration of competing interest

The authors declare that there are no conflicts of interest.
